# Genetics of Cardiovascular Disease: Fishing for Causality

**DOI:** 10.3389/fcvm.2018.00060

**Published:** 2018-06-01

**Authors:** Christoph Paone, Federica Diofano, Deung-Dae Park, Wolfgang Rottbauer, Steffen Just

**Affiliations:** ^1^Molecular Cardiology, Department of Internal Medicine II, University of Ulm, Ulm, Germany; ^2^Department of Internal Medicine II, University of Ulm, Ulm, Germany

**Keywords:** genome-wide association study, zebrafish, functional genomics, CRISPR/Cas9, heart disease

## Abstract

Cardiovascular disease (CVD) is still the leading cause of death in all western world countries and genetic predisposition in combination with traditional risk factors frequently mediates their manifestation. Genome-wide association (GWA) studies revealed numerous potentially disease modifying genetic loci often including several SNPs and associated genes. However, pure genetic association does not prove direct or indirect relevance of the modifier region on pathogenesis, nor does it define within the associated region the exact genetic driver of the disease. Therefore, the relevance of the identified genetic disease associations needs to be confirmed either in monogenic traits or in experimental *in vivo* model system by functional genomic studies. In this review, we focus on the use of functional genomic approaches such as gene knock-down or CRISPR/Cas9-mediated genome editing in the zebrafish model to validate disease-associated genomic loci and to identify novel cardiovascular disease genes. We summarize the benefits of the zebrafish for cardiovascular research and highlight examples demonstrating the successful combination of GWA studies and functional genomics in zebrafish to broaden our knowledge on the genetic and molecular underpinnings of cardiovascular diseases.

## Introduction

Cardiovascular disease (CVD) is the leading cause of mortality worldwide. CVD describes a class of diseases affecting the heart and blood vessels, such as cardiomyopathies, coronary artery disease, heart failure or arrhythmias. A variety of risk factors, such as smoking, obesity, hypertension or high cholesterol can be causative for CVD, however, it is understood that these traditional risk factors only contribute to a fraction of disease cases (1). Therefore, researchers also focus on the definition of the genetic basis of CVD to identify disease mechanisms independent of environmental risk factors. Recent advances in next-generation sequencing (NGS) techniques enable now an unbiased, whole-genome analysis of patients to identify disease-associated genetic alterations. One of these approaches comprises genome-wide association (GWA) studies (GWAS) that have emerged as a powerful tool to identify disease-related loci and have become a valuable candidate resource for disease causing genes and variants. A GWA study is a hypothesis-free approach utilizing the information of hundreds of thousands of genetic variants across the genome, so-called SNPs (single nucleotide polymorphisms), in large population samples. In this context, GWAS findings are purely genetic, but significant associations between SNPs and the disease are therefore excellent starting-points for detailed follow-up studies. More than 10,000 of such significant associations with disorders and genomic traits were reported by GWA studies resulting in new insights into biology and molecular mechanisms of various diseases (2). Online platforms like the GWAS catalog provide researchers collected data of published GWA studies and enable the open-access view into these genome-wide analyses (3). The GWAS catalog comprises studies on a variety of diseases ranging from neurological disorders, various cancer types to cardiac diseases, such as cardiomyopathies or arrhythmias. All GWA studies rely on the exact definition of the disease phenotype in patients to obtain an as specific cohort as possible. The influence of a mixed cohort, secondary disease mechanisms or environmental variations might lead to non-significant or underestimated results. This could be particularly observed for GWA studies focusing on heart failure mechanisms (4). Although well-designed, some GWA studies still lack the clinical relevance due to missing causality of the candidate genes. In order to get a fast and reliable validation of GWAS hits, an adequate experimental model in follow-up studies is fundamental. Several model systems are available, ranging from cell culture to animal models and each model has its pros and cons depending on the respective disease mechanism. During the last decades, the zebrafish (*Danio rerio*) has emerged rapidly as a model organism in cardiovascular research. In this review, we will focus on the use of the zebrafish to investigate the pathomechanisms of heart diseases and discuss its suitability as an experimental tool to validate the disease-association of genes identified by GWA studies.

## The zebrafish: small fish, big impact

Zebrafish possess a variety of features that are advantageous for the use as experimental model organism. Due to their small size (2–4 cm), zebrafish are easy to handle and one female can produce around 200 eggs per week. Zebrafish embryos develop externally and very rapidly to freely swimming and fed larvae within 5 days (5, 6). The zebrafish is an excellent system for microscopic applications as embryos are transparent and numerous transgenic fluorescent reporter lines are available or can easily be produced (7). Such reporter lines are widely used to image organ development and morphology as well as physiological parameters like membrane voltage or calcium transients (8, 9). Because of their suitability for imaging applications, zebrafish are also highly interesting for high-throughput small compound screens. This is enabled by already existing and continuously improving screening platforms e.g., for the automated detection of heartbeat, heartrate and fractional shortening in embryos or isolated hearts of adult zebrafish (10–13). Such set-ups facilitate rapid and high-throughput preclinical tests of large numbers of small molecules and help to identify novel therapeutic strategies (14–17).

Beside the mentioned general advantages, zebrafish exhibit characteristics making them appropriate to study heart development and disease (18–21). Zebrafish heart development proceeds fast and results in a differentiated two-chambered heart within 48 hpf (22). In addition, zebrafish embryos, in contrast to mammalian or avian embryos, are able to cover their oxygen demand by diffusion during the first days of development and are not dependent on blood circulation. This enables the investigation of gene knockouts or knockdowns, even if they lead to severe defects of the cardiovascular system (23).

On a genetic basis, humans and zebrafish share a 70% sequence similarity and 84% of human disease-causing genes can also be found in the zebrafish genome (24, 25). However, regarding the cardiovascular system, there are basic morphological differences as the zebrafish heart consists of only two heart chambers, one atrium and one ventricle. This is on the one hand advantageous as it displays a simplified experimental model, on the other hand, these anatomical difference may limit the translation of findings into the mammalian system (21). Unlike mammals, which develop a coronary system during embryogenesis, zebrafish show a vasculature on the heart surface starting at 1–2 month post hatching (26). This restricts the study of coronary artery disease (CAD) to adult zebrafish, although it is possible to analyze basic mechanisms of atherosclerotic lesion development also in the vasculature of zebrafish embryos (27). There are also several parameters of the zebrafish heart that are closer to the human situation than mammalian model organisms, such as the mouse (17). For example, the zebrafish heart rate of 120–180 bpm (beats per min) is comparable to the 60–100 bpm of the human heart, whereas the mouse heart beats 5 times faster. Furthermore, zebrafish ECG parameters are very similar to human values enabling a direct comparison and translation of experimental findings (20, 28).

**Figure 1:**
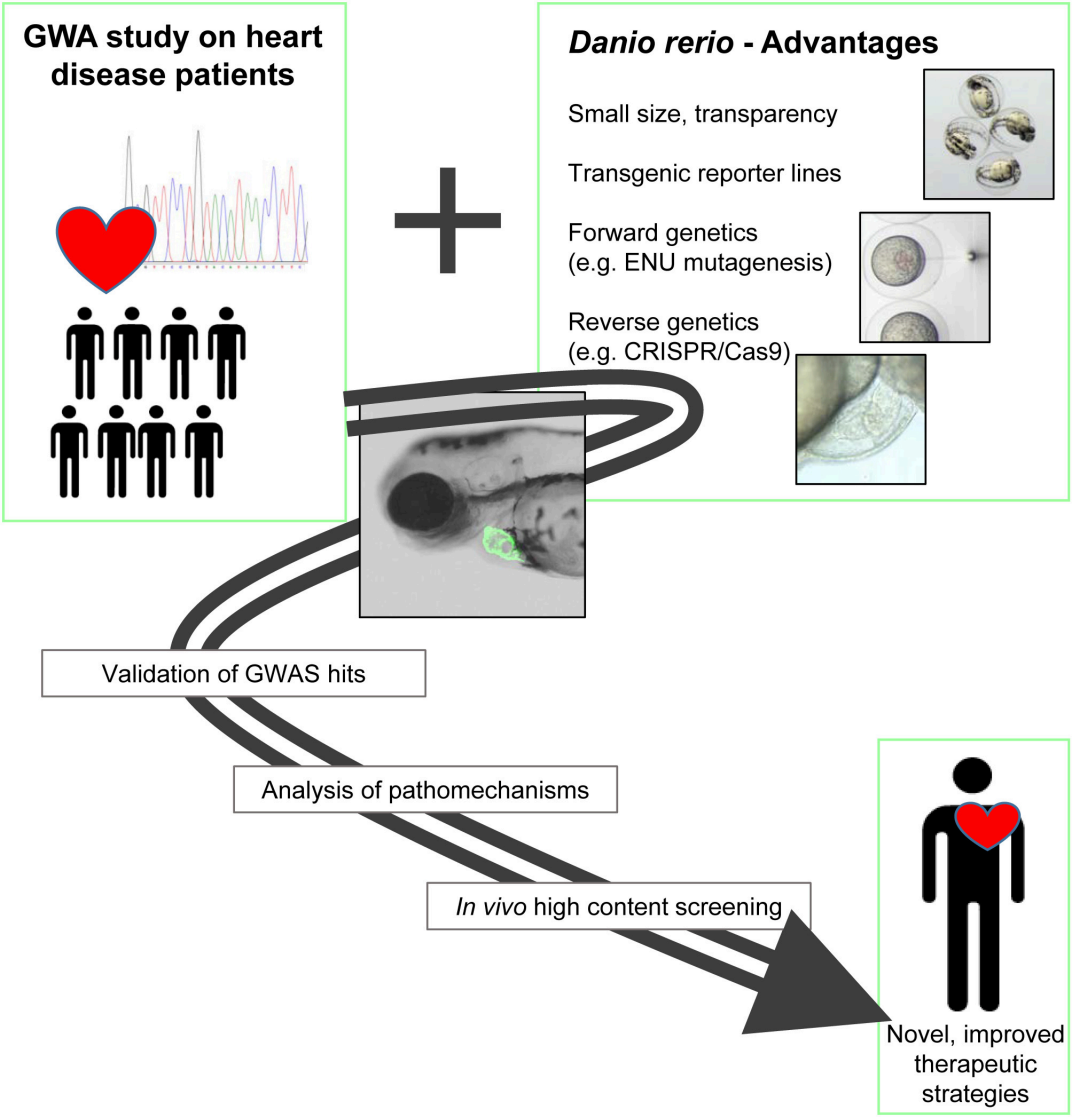
GWAS and zebrafish: a powerful combination for cardiovascular research. The benefits of the zebrafish as an experimental system can enormously help to validate the functional relevance of candidate genes identified by GWA studies. In addition, the system enables the *in vivo* analysis of underlying pathomechanisms and is highly suitable for high throughput screening applications. In summary, the combination of GWAS and the zebrafish experimental system has the potential to lead to improved and specific therapeutic approaches.

In addition to the great benefits of the zebrafish in regard to organ development, physiology, handling and imaging, its suitability for genetic manipulation is another big advantage of the system (Figure 1). Here, we will give a compressed overview on the repertoire of zebrafish genetic tools and highlight examples, where they have been used to demonstrate the causality of genes or loci identified by GWAS.

## Zebrafish genetic screens: a (swimming) pool of disease genes

Before GWAS data became more and more accessible, candidate driven approaches have been very successful in identifying disease-associated mutations. Here, known molecular players and/or regulators of a specific disease-related pathway are screened in a cohort or hereditary trait to find an association with the pathological outcome. Forward genetic screens in zebrafish contributed a lot to these studies as a variety of genes responsible for cardiovascular defects were identified by zebrafish mutant lines arising from mutagenesis screens (18, 29). These screens, comparable to GWAS, have the advantage to be hypothesis-free approaches that identify genetic mutations via a randomly induced phenotype. The most prominent mutagenesis screens are based on alkylating agents like N-ethyl-N-nitrosurea (ENU), which give rise to point mutations leading to nonsense or missense mutations that affect the regulatory and coding region of genes (30–32).

Although mainly recessive and single inherited mutations can be analyzed in such mutagenesis screens, the combination of zebrafish forward genetics followed by human genome analysis led to the identification of several disease-related genes. One example is the zebrafish mutant *main squeeze* (*msq*), which harbors a mutation in the gene encoding ILK (Integrin-linked kinase). *Msq* mutants display progressive loss of ventricular contractility leading to heart failure (33). Another ILK mutant line, *lost contact* (*loc*), also displays a cardiomyopathy phenotype (34). After identifying ILK mutations as causative for the *loc* mutant phenotype, Knöll et al. performed a mutation screen in the ILK gene of human cardiomyopathy patients. This screen revealed an ILK mutation that was associated with the disease and its disease causing effect could be again validated in *loc* mutants. These examples show that zebrafish can serve as (I) a resource for new candidate genes in heart failure through forward genetic screens as well as (II) a model organism to validate potential disease-causing mutations in reverse genetic analyses.

## Reverse genetic approaches in zebrafish

Reverse genetics can be regarded as targeted investigation of a gene of interest by increasing, reducing or silencing its expression. A diversity of reverse genetic tools can be applied in zebrafish, however, several characteristics of zebrafish genetics have to be kept in mind. Zebrafish underwent a whole-genome duplication event with the consequence that for many genes a partially redundant paralog is present (24, 35). In addition, there often exist several transcripts of the same gene and the knockdown or knockout of several genes might be necessary to model the loss-of-function phenotype of a human ortholog. Another aspect that needs to be considered is the genetic variation between and within zebrafish strains that might have an impact on the phenotype and the conclusion drawn from functional analyses (36).

An important and helpful resource for reverse genetic investigations is the zebrafish mutant project that provides a growing list of fish lines with a defined mutation in a specific gene (25). These mutations are induced by chemical mutagenesis, similar to the one used in forward genetic screens, and identified by high-throughput DNA genotyping, an approach called TILLING (targeting induced local lesions in genomes) (37, 38). If a desired and appropriate mutation is available, this open source platform might give scientists a fast access to a loss-of-function model that can be directly used for functional studies. The reverse genetic tools that can be applied in zebrafish are mainly (A) mRNA overexpression and (B) transgenesis, (C) Morpholino-modified antisense oligonucleotide (MO) mediated knockdown or (D) genome editing techniques such as ZFNs (zinc finger nucleases), TALENs (transcription activator-like effector nucleases) or the CRISPR/Cas9 system (clustered regulatory interspaced short palindromic repeats).

### mRNA overexpression

Injecting synthetic capped mRNA encoding the protein of interest into early embryonic stages is commonly used as a standard method to induce transient overexpression of genes or gene variants (e.g., SNPs/variants identified in GWAS or next-generation sequencing) for gain-of-function or loss-of-function studies. Thus, mRNA overexpression in zebrafish was used for example to analyze mutations in the *NEXN* (Nexilin) gene that were identified in human DCM (dilated cardiomyopathy) patients (39). When overexpressed in zebrafish embryos, these mutant *NEXN* variants induced a severe DCM phenotype showing the suitability of the method for fast and effective testing of the impact of putative mutations. Even though mRNA overexpression is an effective way to elucidate functions of specific genes, its use is restricted to focus on early organ development and function because of limited stability of the injected mRNA.

### Transgenesis

Transgenesis in zebrafish involves the insertion of foreign DNA into the genome and is often used to create reporter lines, in which a fluorescent reporter gene under the control of a specific promoter is used to label a particular tissue, organ or cell type. The most commonly used system to insert a transgene in the zebrafish germline is the *Tol2* system derived from medaka fish. This autonomously active *Tol2* element harbors a gene that encodes for a transposase mediating the transposition of the *Tol2* element into the genome (40). For transgenesis of zebrafish, the sequence or gene of interest needs to be flanked by 150–200 bp ends of the *Tol2* element. Injection of this construct together with i*n vitro* transcribed transposase mRNA leads to the highly efficient generation of transgenic F_1_ offspring (41, 42). For zebrafish heart development and function a variety of transgenic lines are present, such as *cmlc2*- (*myosin light chain 7, myl7*) promoter driven reporter lines that specifically label cardiomyocytes of both heart chambers (43, 44). In addition, random insertional transgenesis of EGFP, so called enhancer trap, was shown to result in various reporter lines specifically labeling cardiac structures (45). A powerful combination is the use of transgenic lines in cell transplantation experiments that are widely-used in zebrafish embryos to investigate cell-autonomous mechanisms. With this approach, Sawamiphak and colleagues could, for example, analyze fusion events between cardiomyocytes during heart development that enable exchange of mRNA or proteins between individual cells (46).

Furthermore, stable transgenic expression of gene variants associated with heart diseases can serve as an appropriate *in vivo* model to study the underlying pathology. Huttner and coworkers, for example, showed that transgenic expression of the D1275N mutation of the human cardiac sodium channel (SCN5A), which is associated with cardiac abnormalities in humans, also leads to bradycardia and defects of the cardiac conduction-system in zebrafish (47). Further developments of transgenesis techniques in regard to tissue specificity or inducibility will broaden the possibilities for transgenesis in zebrafish and help to create improved experimental systems for cardiac research (48, 49).

### Morpholino-mediated knockdown

Morpholinos (MO) are knockdown reagents that are very stable, resistant to nucleases and can be injected into 1-cell stage zebrafish embryos. Thus, they became a standard approach for gene knockdown in zebrafish (50, 51). In a variety of cardiovascular research studies, MO-mediated knockdowns were performed to analyze the disease association of a particular gene and/or to model specific pathological features. For example, knockdown of genes that are associated with DCM progression in humans also results in cardiomyopathy in the zebrafish (39, 52). However, phenotypes induced by MOs may be more severe than those of the corresponding mutants. This discrepancy can be a result of genetic compensation in the mutant or due to off-target effects of the used MO (53–55). Therefore, proper control of MO specificity, efficiency and toxicity should be performed in all applications (51).

### Genome editing techniques

Genome editing has evolved as a major strategy to disrupt the coding sequence of genes of interest leading to a loss-of function. During recent years, various CRISPR/Cas9, ZFN and TALEN approaches were developed and applied in zebrafish research to create gene knockouts. The detailed technical aspects are not the focus of this review, but are reviewed elsewhere (56, 57). ZFNs and TALEN approaches were successfully applied in zebrafish cardiovascular research studies (58, 59). For instance, ZFN-mediated knockout of *GATA2* results in severe defects in vascular organization highlighting the importance of this gene for cardiovascular development (60).

The discovery of CRISPR/Cas9 as a genome editing method declared a new era of reverse genetics (61, 62). The CRISPR/Cas9 system is the most efficient genome editing method for reverse genetics in zebrafish and exhibits, due to its simplicity and applicability, many advantages compared to ZFN and TALENs (63). The CRISPR/Cas9 system is a two-component complex composed of the Cas9 endonuclease, which induces DSBs (double-strand break) and a guide RNA (gRNA) recognizing specific DNA sequences (62). CRISPR/Cas9 is remarkably simple and adaptable due to its unique mechanism and therefore, is chosen as a major genome-editing tool among all the technologies present in the zebrafish field (64). Its suitability for reverse genetics in zebrafish, in regard to cardiovascular research, could be shown for example by a knockout of the large transcript *pr130* of the Protein Phosphatase 2 Regulatory subunit Bα (PPP2R3A) (65). Here, two *pr130* knockout lines demonstrated the importance of *pr130* for cardiac development and function and provide a suitable genetic model to study the underlying pathomechanisms. An aspect that needs to be considered in all genome editing approaches is the possible presence of off-target effects. Unbiased whole genome analyses of CRISPR/Cas9 off-target effects are still missing and researchers are most often restricted to the analysis of off-target genes that are predicted by computational approaches (66). By careful design and selection of gRNA sequences and the use of nuclease variants with high specificity the risk for off-target effects can be minimized. Additionally, continuous outcrossing of the mutation and the comparison of at least two independently produced knockout lines help to prevent misinterpretations of a genotype-phenotype connection.

Recently, a variety of improvements and new applications of the CRISPR/Cas9 system evolved contributing to the fast implementation of the method in many zebrafish laboratories. The classical targeted knock-out strategy involves the injection of gRNA and Cas9 (mRNA or protein) into 1-cell stage embryos and the screening for germline mutations in subsequent generations (67). Another strategy uses e.g., a catalytically dead Cas9 protein (dCas) lacking endonuclease activity to generate a DNA recognition complex that can specifically perturb transcriptional elongation, RNA polymerase binding, or transcription factor binding (68). CRISPR/Cas9 can also be used to generate defined knock-in fish lines with integrated SNPs, stop codons, HA tags, loxP sites or fluorescent proteins (69, 70). The CRISPR/Cas9 toolbox is continuously growing and recent progress is achieved by using this method for tissue-specific blockage of gene function (71, 72) or by combining the strategy with optogenetic tools to have temporal control over Cas9 activity (73). In the context of cardiovascular research, these improvements will help to obtain heart-specific knockouts and to mimic the late onset and slow progression of many cardiomyopathy subtypes.

## GWA studies and functional genomics in zebrafish: a powerful combination

The zebrafish functional genomics toolbox enables a defined analysis of theoretically any gene of interest *in vivo*. This makes the zebrafish a valuable experimental platform to validate putative disease causing genes that are identified by GWA studies. Indeed, a variety of genome-wide surveys, focusing on heart diseases, already used zebrafish to prove their initial findings. Table 1 summarizes selected examples of genome-wide studies, for which the resulting candidate genes could be confirmed by zebrafish reverse genetics. An early GWA study in 2008 identified three co-segregating genes (*HBEGF, IK*, and *SRA1*) associated with DCM (87). For Heparin-binding EGF-like growth factor (*HBEGF*), the linkage to DCM progression was already known from mouse knockout studies (88). The DCM-association for the cytokine IK and the steroid receptor RNA activator1 SRA1 is a new connection arising from this study. The disease-relevance of these candidate genes could be verified in zebrafish embryos. The MO-mediated knockdown of all three genes, HBEGF, SRA1 and IK resulted in severe pericardial edema, accompanied by reduced fractional shortening (FS) of the ventricular chamber (87). Another study focused on the genetic basis of CAID (Chronic atrial and intestinal dysrhythmia) and found a linkage to the *SGOL1* gene (Shugosin-like 1) (86). The authors could show that *SGOL1* is expressed in the sinoatrial region and atrioventricular valves of the adult zebrafish heart. Consistent with its expression pattern, the knockdown of *SGOL1* in zebrafish embryos resulted in bradycardia confirming the involvement of SGOL1 in heart rhythm control (86). *KCNIP1* (potassium voltage-gated channel interacting protein 1) is another example of a gene that could be linked by whole genome analysis to heart disease, here atrial fibrillation (AF) (82). Interestingly, the reported mutation does not lead to a loss of function, but is suggested to increase KCNIP1 levels. The authors modeled this by the overexpression of *KCNIP1* in zebrafish and could show that increased KCNIP1 levels can result in transient atrial tachycardia and AF during high-rate pacing (82). Norton et al. (79) identified *BAG3* (Bcl-2 associated anthanogene 3) as a DCM-associated gene and could confirm its disease relevance by knocking-down *BAG3* in zebrafish embryos. BAG3-deficient fish showed severe pericardial edemas and a decreased fractional shortening as well as a reduced peak blood cell flow velocity (79). A second study independently identified also *BAG3* as a potential DCM-causing gene (78). In addition, the functional requirement of BAG3 for heart as well as skeletal muscle function was also confirmed by an independent MO-based analysis of several myopathy-related genes (80). Another gene linked to DCM that was identified by a whole genome study is Filamin C (*FLNC*) (81). The authors of this study also used MO-knockdown experiments to validate their findings. *FLNC* morphants exhibited dysmorphic or dilated heart chambers as well as impaired heart looping confirming the importance of FLNC for heart function and development (81). Lundby et al. (83) used a GWA approach combined with tissue-specific proteomics to analyze genes associated with LQTS (Long QT Syndrome) (83). They could identify Vinculin (*VCL*) as a disease-associated gene and could confirm its relevance by using a *VCL-*knockdown approach in zebrafish. In these experiments, the authors measured cardiac repolarization in isolated embryonic hearts using fluorescent probes and could observe an impaired repolarization response upon loss of VCL (83). Additionally, by using a gene-trap mutant zebrafish line as well as a CRISPR/Cas9 knockout line of *VCL*b, Cheng et al. could confirm its disease-relevance (85) and a MO-based knockdown of *VCL* in zebrafish in another independent approach also validated the role of Vinculin in heart function and structure (84).

**Table 1:** Genome-Wide Studies using the zebrafish model to validate the causality of candidate genes.

**Genome-wide study**	**Disease**	**Gene(s)**	**Zebrafish genetic tool**	**Zebrafish phenotype**	**References**
(74): GWAS in 1,910 patients/3,630 controls	DCM	*HSPB7*	MO knockdown TALEN knockout	Heart looping defects Increased susceptibility to cardiomyopathy	(75, 76)
(77): GWAS in 1,590 patients/577 controls	HF	*HSPB7*			
(78): GWAS in 1,179 patients/1,108 controls	DCM	*BAG3*	MO knockdown	Cardiac and skeletal myopathy	(79, 80)
(79): WES in one multigeneration family	DCM	*BAG3*			
(81): WES in three multigeneration families	DCM	*FLNC*	MO knockdown	Abnormal cardiac function and structure	Same study (80)
(82): WES on three unrelated probands	AF	*KCNIP1*	Overexpression MO knockdown	Overexpression promotes AF	Same study
(83): Combined genetic and proteomic GWAS of 4 cohorts with 17,692 samples	LQTS	*VCL*	MO knockdown Gene-Trap CRISPR/Cas9	Defective cardiac repolarization Myocardial contractile dysfunction	Same study (84, 85)
(86): WES on three cases	CAID	*SGOL1*	MO knockdown	Bradycardia	Same study
(87): GWAS in 590 patients/732 controls	DCM	*HBEGF, IK, SRA1*	MO knockdown	Myocardial contractile dysfunction	Same study

*Mentioned here are examples found by manual database analysis. The list might not be exhaustive*.

*AF, atrial fibrillation; CAID, Chronic atrial and intestinal dysrhythmia; DCM, dilated cardiomyopathy; GWAS, genome-wide association study; HF, heart failure; LQTS, long QT syndrome; MO, morpholino; WES, whole exon sequencing*.

Other studies did not use the zebrafish to validate their candidate genes, however, independent follow-up studies could confirm the disease-causing potential of some of them. For example, two independent GWA studies on DCM and heart failure identified SNPs near the *HSPB7* (Heat Shock Protein Family B Member 7) gene to be associated with the disease (74, 77). Three years later, Rosenfeld et al. could confirm the requirement of HSPB7 in heart function and structure by MO-based knockdown experiments. HSPB7 depletion led to impaired cardiac morphogenesis due to defects in ventricular size, but also due to an early block of heart tube formation (75). By using a TALEN-mediated knockout of *HSPB7*, the same group recently showed that loss of *HSPB7* increases the susceptibility of adult mutant zebrafish for cardiomyopathy due to impaired protein homeostasis serving as another proof of the initial GWAS findings (76). In addition, this zebrafish study and most of the above mentioned, are not only validating candidate genes from GWA studies, they also allow a more detailed investigation of the underlying pathomechanisms and help to identify novel disease-associated pathways and protein networks.

## Conclusion and future perspectives

The zebrafish has a variety of advantages to be combined with GWAS. Zebrafish are easy to keep, to handle and to image, show many physiological and genetic similarities to humans and are highly suitable for genetic manipulations. These features help to establish valid disease models and allow a plethora of follow-up studies.

Due to obvious differences in morphology and living environment, it should be clear that a simple translation of findings from the zebrafish system to humans is not always possible. In addition, the probably biggest difference and, also peculiarity of the zebrafish heart compared to mammals is the ability to regenerate injury (89). This may result in drawbacks and limitations when comparing pathomechanisms in fish and humans, but also makes the zebrafish a highly interesting model to study the underlying mechanisms of regeneration (90, 91). It is important to mention that a disease-association of a particular gene that cannot be confirmed in zebrafish doesn't necessarily mean that it is not causative for the phenotype. Mechanisms like intrinsic repair processes or genetic compensation may hide a causative effect of a gene mutation. In such situations, other experimental models, like rodents or patient-derived iPSC (induced pluripotent stem cells) might lead to clearer results (92, 93). Nevertheless, all mentioned benefits make the zebrafish a valid and highly suitable model to investigate cardiovascular pathologies and to prove findings from GWAS. Many SNPs identified in GWA studies are located in non-coding regions of the genome and might affect for example enhancer or repressor binding, microRNA binding sites or chromatin structure. Also for these kinds of mutations, the zebrafish system can help to identify their *in vivo* relevance, biological role and the underlying pathomechanisms. Madelaine et al. (94), for example, could very recently confirm human disease-associated SNPs in CNEs (conserved non-exonic elements) by using CRISPR/Cas9-mediated deletion of the respective non-coding locus (94).

We are sure that the fruitful synergism between GWAS and zebrafish in cardiac research will expand in the future and will lead to the identification of novel disease-causing genes and variants and help to screen for possible therapeutic strategies.

## Author contributions

CP, FD, D-DP, WR, and SJ contributed substantially to the conception, drafting, and revision of the manuscript and approved the final version.

### Conflict of interest statement

The authors declare that the research was conducted in the absence of any commercial or financial relationships that could be construed as a potential conflict of interest.

## References

[1] Thanassoulis G Vasan RS. Genetic cardiovascular risk prediction. Circulation (2010) 122:2323. 10.1161/CIRCULATIONAHA.109.909309 21147729 PMC3075800

[2] Visscher PM Wray NR Zhang Q Sklar P McCarthy MI Brown MA. 10 Years of GWAS discovery: biology, function, and translation. Am J Hum Genet (2017) 101:5–22. 10.1016/j.ajhg.2017.06.005 28686856 PMC5501872

[3] MacArthur J Bowler E Cerezo M Gil L Hall P Hastings E. The new NHGRI-EBI catalog of published genome-wide association studies (GWAS Catalog). (2017). Nucleic Acids Res 45:D896–901. 10.1093/nar/gkw1133 27899670 PMC5210590

[4] Rau CD Lusis AJ Wang Y. Genetics of common forms of heart failure: challenges and potential solutions. Curr Opin Cardiol. (2015) 30:222–7. 10.1097/HCO.0000000000000160 25768955 PMC4406340

[5] Cunliffe VT Zebrafish: a practical approach. Edited by NÜSSLEIN-VOLHARD C. DAHM R. Oxford University Press 2002. 322 pages. ISBN 0 19 963808 X. Genetical Research (2003) 82:79 10.1017/S0016672303216384

[6] Link BA Megason S G (2008). Zebrafish as a Model for Development. Sourcebook of Models for Biomedical Research. P. M. Conn. Totowa, NJ: Humana Press.

[7] Weber M Huisken J. *In vivo* imaging of cardiac development and function in zebrafish using light sheet microscopy. Swiss Med Wkly. (2015). 145:w14227. 10.4414/smw.2015.14227 26700795

[8] Russell JT. Imaging calcium signals *in vivo*: a powerful tool in physiology and pharmacology. Br J Pharmacol (2011) 163:1605–25. 10.1111/j.1476-5381.2010.00988.x 20718728 PMC3166690

[9] Hou JH Kralj JM Douglass AD Engert F Cohen AE. Simultaneous mapping of membrane voltage and calcium in zebrafish heart *in vivo* reveals chamber-specific developmental transitions in ionic currents. Front Physiol (2014) 5:344. 10.3389/fphys.2014.00344 25309445 PMC4161048

[10] Kitambi SS Nilsson ES Sekyrova P Ibarra C Tekeoh GN Andäng M. Small molecule screening platform for assessment of cardiovascular toxicity on adult zebrafish heart. BMC Physiol (2012) 12:3. 10.1186/1472-6793-12-3 22449203 PMC3334682

[11] Spomer W Pfriem A Alshut R Just S Pylatiuk C. High-throughput screening of zebrafish embryos using automated heart detection and imaging. J Lab Auto (2012) 17:435–42. 10.1177/2211068212464223 23053930

[12] Pylatiuk C Sanchez D Mikut R Alshut R Reischl M Hirth S. Automatic zebrafish heartbeat detection and analysis for zebrafish embryos. Zebrafish (2014) 11:379–83. 10.1089/zeb.2014.1002 25003305 PMC4108935

[13] Nasrat S Marcato D Hirth S Reischl M Pylatiuk C Semi-automated detection of fractional shortening in zebrafish embryo heart videos. Curr Direct Biomed Eng. (2016) 2:233 10.1515/cdbme-2016-0052

[14] Mathias JR Saxena MT Mumm JS. Advances in zebrafish chemical screening technologies. Future Med Chem (2012) 4:1811–22. 10.4155/fmc.12.115 23043478 PMC3566566

[15] Asnani A Peterson RT. The zebrafish as a tool to identify novel therapies for human cardiovascular disease. Dis Models Mech (2014) 7:763–7. 10.1242/dmm.016170 24973746 PMC4073266

[16] Keßler M Rottbauer W Just S. Recent progress in the use of zebrafish for novel cardiac drug discovery. Expert Opin Drug Discov (2015) 10:1231–41. 10.1517/17460441.2015.1078788 26294375

[17] Gut P Reischauer S Stainier DY Arnaout RR. Little Fish, Big Data: zebrafish as a model for cardiovascular and metabolic disease. Physiol Rev (2017) 97:889–938. 10.1152/physrev.00038.2016 28468832 PMC5817164

[18] Dahme T Katus H A Rottbauer W Fishing for the genetic basis of cardiovascular disease. Dis Models Mech. (2009) 2:18–22. 10.1242/dmm.000687 19132116 PMC2615162

[19] Bakkers J. Zebrafish as a model to study cardiac development and human cardiac disease. Cardiovasc Res (2011) 91:279–88. 10.1093/cvr/cvr098 21602174 PMC3125074

[20] Pott A Rottbauer W Just S. Functional genomics in zebrafish as a tool to identify novel antiarrhythmic targets. Curr Med Chem. (2014) 21:1320–29. 10.2174/0929867321666131227130218 24372224

[21] Brown RD Samsa AL Qian L Liu J. Advances in the study of heart development and disease using zebrafish. J Cardiovasc Dev Dis. (2016) 3:2. 10.3390/jcdd3020013 27335817 PMC4913704

[22] Kimmel CB Ballard WW Kimmel SR Ullmann B Schilling T F. Stages of embryonic development of the zebrafish. Dev Dyn. (1995) 203:253–310. 10.1002/aja.1002030302 8589427

[23] Stainier DYR. Zebrafish genetics and vertebrate heart formation. Nat Rev Genet. (2001) 2:39. 10.1038/35047564 11253067

[24] Howe K Clark MD Torroja CF Torrance J Berthelot C Muffato M. The zebrafish reference genome sequence and its relationship to the human genome. Nature (2013) 496:498–503. 10.1038/nature12111 23594743 PMC3703927

[25] Kettleborough RNW Busch-Nentwich EM Harvey SA Dooley CM de Bruijn E van Eeden F. (2013). A systematic genome-wide analysis of zebrafish protein-coding gene function. Nature 496:494–7. 10.1038/nature11992 23594742 PMC3743023

[26] Harrison MRM Bussmann J Huang Y Zhao L Osorio A Burns CG. Chemokine guided angiogenesis directs coronary vasculature formation in zebrafish. Dev Cell (2015) 33:442–54. 10.1016/j.devcel.2015.04.001 26017769 PMC4448080

[27] Fang L Liu C Miller Y I. Zebrafish models of dyslipidemia: relevance to atherosclerosis and angiogenesis. Transl Res. (2014) 163:99–108. 10.1016/j.trsl.2013.09.004 24095954 PMC3946603

[28] Wang LW Huttner IG Santiago CF Kesteven SH Yu ZY Feneley M P.. Standardized echocardiographic assessment of cardiac function in normal adult zebrafish and heart disease models. Dis Model Mech. (2017) 10:63–76. 10.1242/dmm.026989 28067629 PMC5278526

[29] Stainier DY Fouquet B Chen JN Warren KS Weinstein BM Meiler SE. Mutations affecting the formation and function of the cardiovascular system in the zebrafish embryo. Development (1996) 123:285. 9007248 10.1242/dev.123.1.285

[30] Driever W Solnica-Krezel L Schier AF Neuhauss SC Malicki J Stemple DL. A genetic screen for mutations affecting embryogenesis in zebrafish. Development (1996) 123:37. 9007227 10.1242/dev.123.1.37

[31] Haffter P Granato M Brand M Mullins MC Hammerschmidt M Kane DA.. The identification of genes with unique and essential functions in the development of the zebrafish, *Danio rerio*. Development (1996) 123:1. 9007226 10.1242/dev.123.1.1

[32] Patton EE Zon LI. The art and design of genetic screens: zebrafish. Nat Rev Genet. (2001) 2:956–66. 10.1038/35103567 11733748

[33] Bendig G Grimmler M Huttner IG Wessels G Dahme T Just S. Integrin-linked kinase, a novel component of the cardiac mechanical stretch sensor, controls contractility in the zebrafish heart. Genes Dev. (2006) 20:2361–72. 10.1101/gad.1448306 16921028 PMC1560411

[34] Knöll R Postel R Wang J Krätzner R Hennecke G Vacaru AM. Laminin-α4 and integrin-linked kinase mutations cause human cardiomyopathy via simultaneous defects in cardiomyocytes and endothelial cells. Circulation (2007) 116:515. 10.1161/CIRCULATIONAHA.107.689984 17646580

[35] Meyer A Schartl M. Gene and genome duplications in vertebrates: the one-to-four (-to-eight in fish) rule and the evolution of novel gene functions. Curr Opin Cell Biol. (1999) 11:699–704. 10.1016/S0955-0674(99)00039-3 10600714

[36] Brown KH Dobrinski KP Lee AS Gokcumen O Mills RE Shi X. Extensive genetic diversity and substructuring among zebrafish strains revealed through copy number variant analysis. Proc Natl Acad Sci USA. (2012) 109:529–54. 10.1073/pnas.1112163109 22203992 PMC3258620

[37] Wienholds E van Eeden F Kosters M Mudde J Plasterk RHA Cuppen E. Efficient target-selected mutagenesis in zebrafish. Genome Res. (2003) 13:2700–07. 10.1101/gr.1725103 14613981 PMC403812

[38] Stemple DL. TILLING — a high-throughput harvest for functional genomics. Nature Rev Genet. (2004) 5:145. 10.1038/nrg1273 14726927

[39] Hassel D Dahme T Erdmann J Meder B Huge A Stoll M. Nexilin mutations destabilize cardiac Z-disks and lead to dilated cardiomyopathy. Nat Med. (2009) 15:1281–8. 10.1038/nm.2037 19881492

[40] Kawakami K Koga A Hori H Shima A. Excision of the Tol2 transposable element of the medaka fish, *Oryzias latipes*, in zebrafish, *Danio rerio*. Gene (1998) 225:17–22. 10.1016/S0378-1119(98)00537-X 9931412

[41] Kawakami K Shima A Kawakami N. Identification of a functional transposase of the Tol2 element, an Ac-like element from the Japanese medaka fish, and its transposition in the zebrafish germ lineage. Proc Natl Acad Sci USA (2000) 97:11403–8. 10.1073/pnas.97.21.11403 11027340 PMC17212

[42] Suster ML Kikuta H Urasaki A Asakawa K Kawakami K (2009). Transgenesis in Zebrafish with the Tol2 Transposon System. Transgenesis Techniques: Principles and Protocols. E. J. Cartwright. Totowa, NJ: Humana Press 10.1007/978-1-60327-019-9_3 19504063

[43] Rottbauer W Saurin AJ Lickert H Shen X Burns CG Wo ZG. Reptin and pontin antagonistically regulate heart growth in zebrafish embryos. Cell (2002) 111:661–672. 10.1016/S0092-8674(02)01112-1 12464178

[44] Rottbauer W Wessels G Dahme T Just S Trano N Hassel D. Cardiac myosin light chain-2. Circ Res. (2006) 99:323. 10.1161/01.RES.0000234807.16034.fe 16809551

[45] Poon KL Liebling M Kondrychyn I Garcia-Lecea M Korzh V. Zebrafish cardiac enhancer trap lines: new tools for *in vivo* studies of cardiovascular development and disease. Dev Dyn. (2010) 239:914–26. 10.1002/dvdy.22203 20063419

[46] Sawamiphak S Kontarakis Z Filosa A Reischauer S Stainier DYR. Transient cardiomyocyte fusion regulates cardiac development in zebrafish. Nat Commun. (2017) 8:1525. 10.1038/s41467-017-01555-8 29142194 PMC5688123

[47] Huttner IG Trivedi G Jacoby A Mann SA Vandenberg JI Fatkin D. A transgenic zebrafish model of a human cardiac sodium channel mutation exhibits bradycardia, conduction-system abnormalities and early death. J Mol Cell Cardiol. (2013) 61:123–32. 10.1016/j.yjmcc.2013.06.005 23791817

[48] Knopf F Schnabel K Haase C Pfeifer K Anastassiadis K Weidinger G. Dually inducible TetON systems for tissue-specific conditional gene expression in zebrafish. Proc Natl Acad Sci USA. (2010) 107:19933–8. 10.1073/pnas.1007799107 21041642 PMC2993373

[49] Akerberg AA Stewart S Stankunas K. Spatial and temporal control of transgene expression in zebrafish. PLoS ONE (2014) 9:e92217. 10.1371/journal.pone.0092217 24643048 PMC3958484

[50] Nasevicius A Ekker SC. Effective targeted gene 'knockdown' in zebrafish. Nat Genet. (2000) 26:216–20. 10.1038/79951 11017081

[51] Stainier DYR Raz E Lawson ND Ekker SC Burdine RD Eisen JS. Guidelines for morpholino use in zebrafish. PLoS Genet. (2017) 13:e1007000. 10.1371/journal.pgen.1007000 29049395 PMC5648102

[52] Vogel B Meder B Just S Laufer C Berger I Weber S. *In-vivo* characterization of human dilated cardiomyopathy genes in zebrafish. Biochem Biophys Res Commun. (2009) 390:516–22. 10.1016/j.bbrc.2009.09.129 19800866

[53] Kok FO Shin M Ni CW Gupta A Grosse AS van Impel A. Reverse genetic screening reveals poor correlation between morpholino-induced and mutant phenotypes in zebrafish. Dev Cell (2015) 32:97–108. 10.1016/j.devcel.2014.11.018 25533206 PMC4487878

[54] Novodvorsky P Watson O Gray C Wilkinson RN Reeve S Smythe C klf2ash317 mutant zebrafish do not recapitulate morpholino-induced vascular and haematopoietic phenotypes. PLoS ONE (2015) 10:e0141611 10.1371/journal.pone.0141611 26506092 PMC4624238

[55] Rossi A Kontarakis Z Gerri C Nolte H Hölper S Krüger M Genetic compensation induced by deleterious mutations but not gene knockdowns. Nature (2015) 524:230–3. 10.1038/nature14580 26168398

[56] Koester R Sassen WA A molecular toolbox for genetic manipulation of zebrafish. Adv Genom Genet. (2015) 5:151–163. 10.2147/AGG.S57585

[57] Sertori R Trengove M Basheer F Ward AC Liongue C. Genome editing in zebrafish: a practical overview. Brief Funct Genomics (2016) 15:322–30. 10.1093/bfgp/elv051 26654901

[58] Huang P Xiao A Zhou M Zhu Z Lin S Zhang B. Heritable gene targeting in zebrafish using customized TALENs. Nat Biotechnol. (2011) 29:699–700. 10.1038/nbt.1939 21822242

[59] Zu Y Tong X Wang Z Liu D Pan R Li Z. TALEN-mediated precise genome modification by homologous recombination in zebrafish. Nat Methods (2013) 10:329–31. 10.1038/nmeth.2374 23435258

[60] Zhu C Smith T McNulty J Rayla AL Lakshmanan A Siekmann AF. Evaluation and application of modularly assembled zinc-finger nucleases in zebrafish. Development (2011) 138:4555–64. 10.1242/dev.066779 21937602 PMC3177320

[61] Jinek M Chylinski K Fonfara I Hauer M Doudna JA Charpentier E. A programmable dual-RNA-guided DNA endonuclease in adaptive bacterial immunity. Science (2012) 337:816–21. 10.1126/science.1225829 22745249 PMC6286148

[62] Wright AV Nunez JK Doudna JA. Biology and applications of CRISPR systems: harnessing nature's toolbox for genome engineering. Cell (2016) 164:29–44. 10.1016/j.cell.2015.12.035 26771484

[63] Hsu PD Lander ES Zhang F. Development and applications of CRISPR-Cas9 for genome engineering. Cell (2014) 157:1262–78. 10.1016/j.cell.2014.05.010 24906146 PMC4343198

[64] Li M Zhao L Page-McCaw PS Chen W. Zebrafish genome engineering using the CRISPR-Cas9 system. Trends Genet. (2016) 32:815–27. 10.1016/j.tig.2016.10.005 27836208 PMC5127170

[65] Yang J Li Z Gan X Zhai G Gao J Xiong C. Deletion of Pr130 interrupts cardiac development in zebrafish. Int J Mol Sci. (2016) 17:1746. 10.3390/ijms17111746 27845735 PMC5133774

[66] Zischewski J Fischer R Bortesi L. Detection of on-target and off-target mutations generated by CRISPR/Cas9 and other sequence-specific nucleases. Biotechnol Adv. (2017) 35:95–104. 10.1016/j.biotechadv.2016.12.003 28011075

[67] Gagnon JA Valen E Thyme SB Huang P Akhmetova L Pauli A. Efficient mutagenesis by Cas9 protein-mediated oligonucleotide insertion and large-scale assessment of single-guide RNAs. PLoS ONE (2014) 9:e98186. 10.1371/journal.pone.0098186 24873830 PMC4038517

[68] Qi LS Larson MH Gilbert LA Doudna JA Weissman JS Arkin AP. Repurposing CRISPR as an RNA-guided platform for sequence-specific control of gene expression. Cell (2013) 152:1173–83. 10.1016/j.cell.2013.02.022 23452860 PMC3664290

[69] Li J Zhang BB Ren YG Gu SY Xiang YH Du JL. Intron targeting-mediated and endogenous gene integrity-maintaining knockin in zebrafish using the CRISPR/Cas9 system. Cell Res. (2015) 25:634–7. 10.1038/cr.2015.43 25849248 PMC4423083

[70] Hoshijima K Jurynec MJ Grunwald DJ. Precise editing of the zebrafish genome made simple and efficient. Dev Cell (2016) 36:654–67. 10.1016/j.devcel.2016.02.015 27003937 PMC4806538

[71] Ablain J Durand EM Yang S Zhou Y Zon LI. A CRISPR/Cas9 vector system for tissue-specific gene disruption in zebrafish. Dev Cell (2015) 32:756–64. 10.1016/j.devcel.2015.01.032 25752963 PMC4379706

[72] Wild R Klems A Takamiya M Hayashi Y Strähle U Ando K. Neuronal sFlt1 and Vegfaa determine venous sprouting and spinal cord vascularization. Nat Commun. (2017) 8:13991. 10.1038/ncomms13991 28071661 PMC5234075

[73] Reade A Motta-Mena LB Gardner KH Stainier DY Weiner OD Woo S. TAEL: a zebrafish-optimized optogenetic gene expression system with fine spatial and temporal control. Development (2017) 144:345–55. 10.1242/dev.139238 27993986 PMC5394756

[74] Stark K Esslinger UB Reinhard W Petrov G Winkler T Komajda M. Genetic association study identifies HSPB7 as a risk gene for idiopathic dilated cardiomyopathy. PLoS Genet. (2010) 6:e1001167. 10.1371/journal.pgen.1001167 20975947 PMC2958814

[75] Rosenfeld GE Mercer EJ Mason CE Evans T. Small heat shock proteins Hspb7 and Hspb12 regulate early steps of cardiac morphogenesis. Dev Biol. (2013) 381:389–400. 10.1016/j.ydbio.2013.06.025 23850773 PMC3777613

[76] Mercer EJ Lin YF Cohen-Gould L Evans T. Hspb7 is a cardioprotective chaperone facilitating sarcomeric proteostasis. Dev Biol. (2018) 435:41–55. 10.1016/j.ydbio.2018.01.005 29331499 PMC5818303

[77] Cappola TP Li M He J Ky B Gilmore J Qu L. Common variants in HSPB7 and FRMD4B associated with advanced heart failure. Circulation (2010) 3:147–54. 10.1161/CIRCGENETICS.109.898395 20124441 PMC2957840

[78] Villard E Perret C Gary F Proust C Dilanian G Hengstenberg C. A genome-wide association study identifies two loci associated with heart failure due to dilated cardiomyopathy. Eur Heart J. (2011) 32:1065–76. 10.1093/eurheartj/ehr105 21459883 PMC3086901

[79] Norton N Li D Mark Rieder J Jill Siegfried D Rampersaud E Züchner S. Genome-wide studies of copy number variation and exome sequencing identify rare variants in BAG3 as a cause of dilated cardiomyopathy. Am J Hum Genet. (2011) 88:273–82. 10.1016/j.ajhg.2011.01.016 21353195 PMC3059419

[80] Bührdel JB Hirth S Keßler M Westphal S Forster M Manta L. *In vivo* characterization of human myofibrillar myopathy genes in zebrafish. Biochem Biophys Res Commun. (2015) 461:217–23. 10.1016/j.bbrc.2015.03.149 25866181

[81] Begay RL Tharp CA Martin A Graw SL Sinagra G Miani D. FLNC gene splice mutations cause dilated cardiomyopathy. JACC (2016) 1:344–59. 10.1016/j.jacbts.2016.05.004 28008423 PMC5166708

[82] Tsai TC Hsieh CS Chang SN Chuang EY Ueng KC Tsai CF. Genome-wide screening identifies a KCNIP1 copy number variant as a genetic predictor for atrial fibrillation. Nat Commun. (2016) 7:10190. 10.1038/ncomms10190 26831368 PMC4740744

[83] Lundby A Rossin EJ Steffensen AB Rav Acha M Newton-Cheh C Pfeufer A. Annotation of loci from genome-wide association studies using tissue-specific quantitative interaction proteomics. Nat Methods (2014) 11:868–74. 10.1038/nmeth.2997 24952909 PMC4117722

[84] Hirth S Bühler A Bührdel JB Rudeck S Dahme T Rottbauer W. Paxillin and focal adhesion kinase (FAK) regulate cardiac contractility in the zebrafish heart. PLoS ONE (2016) 11:e0150323. 10.1371/journal.pone.0150323 26954676 PMC4782988

[85] Cheng F Miao L Wu Q Gong X Xiong J Zhang J. Vinculin b deficiency causes epicardial hyperplasia and coronary vessel disorganization in zebrafish. Development (2016) 143:3522–31. 10.1242/dev.132936 27578788

[86] Chetaille P Preuss C Burkhard S Côté JM Houde C Castilloux J. Mutations in SGOL1 cause a novel cohesinopathy affecting heart and gut rhythm. Nat Genet. (2014) 46:1245–9. 10.1038/ng.3113 25282101

[87] Friedrichs F Zugck C Rauch GJ Ivandic B Weichenhan D Muller-Bardorff M. HBEGF, SRA1, and IK: Three cosegregating genes as determinants of cardiomyopathy. Genome Res. (2009) 19:395–403. 10.1101/gr.076653.108 19064678 PMC2661798

[88] Iwamoto R Yamazaki S Asakura M Takashima S Hasuwa H Miyado K. Heparin-binding EGF-like growth factor and ErbB signaling is essential for heart function. Proc Natl Acad Sci USA. (2003) 100:3221–6. 10.1073/pnas.0537588100 12621152 PMC152273

[89] Poss KD Wilson LG Keating MT. Heart regeneration in zebrafish. Science (2002) 298:2188. 10.1126/science.1077857 12481136

[90] Sehring IM Jahn C Weidinger G. Zebrafish fin and heart: what's special about regeneration? Curr Opin Genet Dev. (2016) 40:48–56. 10.1016/j.gde.2016.05.011 27351724

[91] González-Rosa JM Burns CE Burns CG. Zebrafish heart regeneration: 15 years of discoveries. Regeneration (2017) 4:105–23. 10.1002/reg2.83 28979788 PMC5617908

[92] Cox RD Church C D. Mouse models and the interpretation of human GWAS in type 2 diabetes and obesity. Dis Model Mech. (2011) 4:155–64. 10.1242/dmm.000414 21324932 PMC3046087

[93] Warren CR O'Sullivan JF Friesen M Becker CE Zhang X Liu P. Induced pluripotent stem cell differentiation enables functional validation of GWAS variants in metabolic disease. Cell Stem Cell (2017) 20:547–57.e547. 10.1016/j.stem.2017.01.010 28388431

[94] Madelaine R Notwell JH Skariah G Halluin C Chen CC Bejerano G A screen for deeply conserved non-coding GWAS SNPs uncovers a MIR-9-2 functional mutation associated to retinal vasculature defects in human. Nucleic Acids Res. (2018) 46:3517–31. 10.1093/nar/gky166 PMC5909433 29518216

